# Supplementary material for: Determining the feasibility of calculating pancreatic cancer risk scores for people with new-onset diabetes in primary care (DEFEND PRIME): study protocol
Source: BMJ Open. 2024 Jan 22;14(1):e079863. doi: 10.1136/bmjopen-2023-079863 (PMC10806670; doi:10.1136/bmjopen-2023-079863)
Supplement: Author's manuscript [file bmjopen-2023-079863.draft_revisions.pdf]

BMJ Open

BMJ Open is committed to open peer review. As part of this commitment we make the peer review history of every article we publish publicly available.

When an article is published we post the peer reviewers' comments and the authors' responses online. We also post the versions of the paper that were used during peer review. These are the versions that the peer review comments apply to.

The versions of the paper that follow are the versions that were submitted during the peer review process. They are not the versions of record or the final published versions. They should not be cited or distributed as the published version of this manuscript.

BMJ Open is an open access journal and the full, final, typeset and author-corrected version of record of the manuscript is available on our site with no access controls, subscription charges or pay-per-view fees (http://bmjopen.bmj.com).

If you have any questions on BMJ Open's open peer review process please email info.bmjopen@bmj.com

BMJ Open

Determining the feasibility of calculating pancreatic cancer risk scores for people with new-onset diabetes in primary care (DEFEND PRIME): study protocol

Journal:	*BMJ Open*
Manuscript ID	bmjopen-2023-079863
Article Type:	Protocol
Date Submitted by the Author:	13-Sep-2023
Complete List of Authors:	Claridge, Hugh; University of Surrey, School of Health Sciences, Faculty of Health and Medical Sciences; National Physical Laboratory<br>Price, Claire; University of Surrey, School of Health Sciences, Faculty of Health and Medical Sciences,; National Physical Laboratory<br>Ali, Rofique; Tower Hamlets Network 1 Primary Care Network<br>Cooke, Elizabeth; National Physical Laboratory<br>de Lusignan, Simon; University of Oxford, Nuffield Department of Primary Care Health Sciences<br>Harvey-Sullivan, Adam; Tower Hamlets Network 1 Primary Care Network; Queen Mary University of London, Centre for Primary Care, Wolfson Institute of Population Health<br>Hodges, Catherine; Surrey and Sussex Cancer Alliance<br>Khalaf, Natalia; Baylor College of Medicine, Center for Innovations in Quality, Effectiveness, and Safety (IQuESt), Michael E. DeBakey Veterans Affairs Medical Center, Section of Gastroenterology and Hepatology, Department of Medicine<br>O'Callaghan, Dean; Tower Hamlets Network 1 Primary Care Network<br>Stunt, Ali; Pancreatic Cancer Action<br>Thomas, Spencer; National Physical Laboratory<br>Thomson, Joanna; Surrey and Sussex Cancer Alliance<br>Lemanska, Agnieszka; University of Surrey, School of Health Sciences, Faculty of Health and Medical Sciences; National Physical Laboratory
Keywords:	Primary Care < Primary Health Care, Pancreatic disease < GASTROENTEROLOGY, Electronic Health Records, Feasibility Studies, Protocols & guidelines < HEALTH SERVICES ADMINISTRATION & MANAGEMENT

SCHOLARONE™
Manuscripts

Determining the feasibility of calculating pancreatic cancer risk scores for people with new-onset diabetes in primary care (DEFEND PRIME): study protocol

Authors

Hugh Claridge[1,2], Claire A. Price[1,2], Rofique Ali[3], Elizabeth Cooke[2], Simon de Lusignan[4], Adam Harvey-Sullivan[3,5], Catherine Hodges[6], Natalia Khalaf[7], Dean O'Callaghan[3], Ali Stunt[8], Spencer A. Thomas[2], Joanna Thomson[6], Agnieszka Lemanska[1,2]

[1]School of Health Sciences, Faculty of Health and Medical Sciences, University of Surrey, Guildford, UK
[2]National Physical Laboratory, Teddington, UK
[3]Tower Hamlets Network 1 Primary Care Network, London, UK
[4]Nuffield Department of Primary Care Health Sciences, University of Oxford, Oxford, UK
[5]Centre for Primary Care, Wolfson Institute of Population Health, Queen Mary University of London
[6]Surrey and Sussex Cancer Alliance, Guildford, UK
[7]Section of Gastroenterology and Hepatology, Department of Medicine, Baylor College of Medicine, Center for Innovations in Quality, Effectiveness, and Safety (IQuESt), Michael E. DeBakey Veterans Affairs Medical Center, Houston, Texas, USA
[8]Pancreatic Cancer Action, Hampshire, UK
Correspondence to: Hugh Claridge, h.claridge@surrey.ac.uk

Abstract

Introduction

Worldwide, pancreatic cancer has a poor prognosis. Earlier diagnosis may improve survival by enabling curative treatment. However, non-specific symptoms and a lack of suitable biomarkers make this challenging. Statistical and machine learning prediction models using risk factors such as patient demographics, symptoms and blood tests are being developed for clinical use to improve earlier diagnosis. One example is the Enriching New-onset Diabetes for Pancreatic Cancer (ENDPAC) model, which employs patients' age, blood glucose and weight changes to provide pancreatic cancer risk scores. However, ENDPAC has yet to be used in clinical settings. In the United Kingdom (UK), blood tests and weight measurements are routinely collected in primary care which, given primary care's central role in assessing and addressing patients' cancer risk, makes it an ideal setting to assess ENDPAC's feasibility.

Methods and analysis

DEFEND PRIME will be a multi-centre observational study determining the feasibility of extracting data from primary care providers in the UK to calculate ENDPAC scores. After developing the data extraction methods, 20 UK GP practices will provide anonymised data on participants aged 50+ years, with a glycated haemoglobin (HbA1c) test result of ≥ 48 mmol/mol and no previous abnormal HbA1c results. ENDPAC scores will be calculated, and descriptive statistics used to summarise the cohort's demographics and assess data quality. Findings will inform the development of a future clinical study, in which ENDPAC scores will be calculated and participants with elevated scores will be invited for clinical workup.

Ethics and dissemination

For peer review only - http://bmjopen.bmj.com/site/about/guidelines.xhtml

This project has been reviewed by the University of Surrey University Ethics Committee and received a favourable ethical opinion (FHMS 22-23 151 EGA). Study findings will be presented at scientific meetings and published in international peer-reviewed journals. Participating GP practices, clinical leads and policy makers will be provided with summaries of the findings.

Strengths and limitations of this study's methods

- Early computerisation of UK primary care, incorporating linkage to pathology systems combined with pay-for-performance for chronic disease management including diabetes, helps to ensure population-wide data.
- The extraction methods will permit validation of the extracted data by GP practice staff prior to transfer to the research team.
- Using HbA1c results only to define new-onset diabetes means this study is not impacted by the quality of diabetes diagnosis coding in primary care.
- This study will raise awareness of new-onset diabetes' association with pancreatic cancer within the primary care community.
- The study period includes the COVID-19 pandemic, thus the data within this period may not reflect data obtained before or after the pandemic.

Introduction

Pancreatic cancer and early diagnosis

Pancreatic cancer is the seventh leading cause of global cancer deaths, with only 10–20 % of patients diagnosed at a sufficiently early stage for curative intervention.(1,2) Survival can be dramatically improved if diagnosed earlier, at a local rather than distant stage – 37 % versus 3 % 5-year survival rate respectively.(3,4) However, there are multiple barriers to early diagnosis including the non-specific nature of the early symptoms,(5) and lack of suitable diagnostic biomarkers, although advances are being made in this area.(6–12)

As with other health conditions including cancers of other sites,(13) statistical and machine learning clinical prediction models are being developed for clinical use, to facilitate earlier diagnosis of pancreatic cancer, in particular its most common subtype, pancreatic ductal adenocarcinoma.(3,14,15) These models range widely in complexity,(16–20) with the simplest models including only a few variables that can also be routinely collected in primary care, making them potentially feasible for use in this setting.

Primary care's role

In most developed countries, primary care constitutes a key element of healthcare provision; in the UK, 90 % of contacts with the UK's National Health Service (NHS) are through primary care.(21) Primary care providers including general practitioners (GPs), are expected to play a central role in assessing and addressing patients' cancer risk.(22) However, it is estimated that GPs see only one new case of pancreatic cancer every five years(23) and, when combined with the non-specific nature of its early symptoms, detection can be very difficult. Clinical prediction models are therefore of real potential value for these clinicians, especially given these challenges of diagnosing pancreatic cancer in the context of their busy work schedules.

Enriching New-Onset Diabetes for Pancreatic Cancer (ENDPAC) model

The simplicity of the ENDPAC model makes it ideally suited for use in primary care as it only uses patient age, weight change and blood glucose measurements, which are routinely collected.(20,24,25) The model is based on the well-documented association of pancreatic cancer with older age and the

For peer review only - http://bmjopen.bmj.com/site/about/guidelines.xhtml

paradoxical development of diabetes with weight loss.(26–34) It also captures the more rapid onset of glycaemic dysregulation found in pancreatic cancer-related diabetes than found in type 2 diabetes. Due to the clinical diagnosis of diabetes sometimes occurring months or even years after diabetes onset,(35–37) ENDPAC instead uses the glycaemic onset to avoid these potential delays and thereby maximises new-onset diabetes' potential for the early diagnosis of pancreatic cancer.(20) The model has undergone external validation in two separate studies in the United States, which established that ENDPAC demonstrates a reasonable ability to differentiate patients with type 2 diabetes from those with glycaemia-defined diabetes who later develop pancreatic cancer.(24,25) It is for these reasons that this study will investigate ENDPAC's feasibility for use in UK primary care settings.

ENDPAC scores

ENDPAC calculates risk scores that patients have pancreatic cancer, by using their age and changes over time to their weight and blood glucose results. According to the model's developers, a score ≤ 0 has a sufficiently high negative predictive value for pancreatic cancer that those with this score can be deemed as only needing management for type 2 diabetes, given their very low risk of pancreatic cancer. A score ≥ 3 is considered to warrant clinical workup for pancreatic cancer.(20) This is because in the original development study and two subsequent external validation studies, patients with a score ≥ 3 had, respectively, a 3.6 %, 2.0 % and 2.6 % 3-year risk of pancreatic cancer, with sensitivities of 78 %, 63 % and 42 %.(20,25,24) The reduced performance in the external validation studies is unsurprising, as performance is often lower when models are applied to different populations than those used to build the model.(38) Furthermore, Sharma *et al*.(20) suggest that with sufficient additional case review processes, 50 % of false positives can be removed, increasing the 3-year risk of pancreatic cancer for patients with a score ≥ 3 from 3.6 % to 10 %.

Rationale for this feasibility study

Before ENDPAC can be used in clinical practice in the UK, it is important to ascertain in what form the required information to calculate ENDPAC scores will be available in UK primary care records, as this is presently unclear. This study will address this, in addition to other pertinent challenges, such as how the data required to calculate ENDPAC scores can be extracted from the multiple different patient record management software platforms used in UK primary care.(39) Preliminary exploratory work with the stakeholder GPs involved in this study has demonstrated that the two primary systems in use accounting for 85 % of the national market share,(39) EMIS Web by EMIS Health and SystmOne by The Phoenix Partnership (TPP), require the development of different approaches to extract the data needed to calculate ENDPAC scores. The reason for developing the extraction methods centrally and providing these for the GP practice staff to use is threefold: first, to maximise the number of GP practices able to calculate ENDPAC scores as few are likely to have staff specialised in writing data extraction software; second, to minimise the time spent by GP practice staff in extracting the data given their already busy work schedules; and third, to ensure consistency in the extraction methods used by the GP practices, so that there can be confidence in the results from each GP practice.

In addition, as values such as weight are often captured opportunistically, it may be that the required data are unavailable for many patients. Furthermore, the units of measurement used for certain variables such as height, weight and HbA1c may differ between practices, as may data management processes, potentially leading to differing data quality levels. The availability of the data and its quality will directly impact whether ENDPAC scores can be calculated, and therefore this information is of critical importance to learn in advance of considering using ENDPAC in clinical practice. This study will address these potential issues and will establish whether it is feasible to calculate ENDPAC scores using routine data contained within electronic healthcare records in UK primary care.

For peer review only - http://bmjopen.bmj.com/site/about/guidelines.xhtml

Study aim and objectives

The aim is to determine the feasibility of calculating ENDPAC scores for people with new-onset diabetes in UK GP practices.

The objectives are to:

1. Develop data extraction methods for primary care. We will work with software developers and GP practice staff who specialise in primary care computer systems to develop open source software and documented search strategies to enable data extraction.
2. Extract anonymised data for eligible participants from 20 GP practices and evaluate the quality and availability of data.
3. Calculate risk scores and undertake descriptive data analysis. We will report the number of people with ENDPAC scores warranting referral for pancreatic cancer investigations and their clinical and demographic characteristics.

Methods and analysis

Design and setting

Determining the feasibility of calculating pancreatic cancer risk scores for people with new-onset diabetes in primary care (DEFEND PRIME) is a multi-centre cohort study. We will extract anonymised data for people with new-onset diabetes from 20 GP practices in the UK. We will analyse the demographics of the cohort, their ENDPAC scores, and assess the quality and availability of data.

GP practice recruitment

We will use several recruitment strategies:

- Presentations and networking at conferences and meetings attended by clinicians and academics working in the early detection field.
- Newsletters sent by the Pancreatic Cancer Action charity, the Surrey and Sussex Cancer Alliance, the National Institute for Health and Care Research Clinical Research Network and the Royal College of General Practitioners.
- Advertising on social media channels to increase study awareness.
- Dissemination of study information by stakeholders and colleagues through their professional networks.

Practices will enrol by completing a data sharing agreement. Based on an hourly rate of £50 for an estimated seven hours' work to extract the data, each practice will be reimbursed £350.

Participant eligibility

For their data to be included, participants must be at least 50 years old and with new-onset diabetes identified between 1st January 2020 to 31st December 2022. For the purposes of this study, new-onset diabetes will be defined by an abnormal glycaemic test result of HbA1c ≥48 mmol/mol. All prior HbA1c test results for the participants must be below this level.

Data extraction methods

We will develop the data extraction methods with professional software developers and staff in the GP practices who specialise in creating searches in patient record management systems. The extraction methods developed will include software and detailed instructions to enable practice staff to perform the data extraction.

For peer review only - http://bmjopen.bmj.com/site/about/guidelines.xhtml
For peer review only

Table 1 details the data that will be extracted, which is modified from Sharma *et al*.(20) Prior to transfer to the research team, HbA1c, weight and BMI results will be selected according to the priorities defined by Sharma *et al*.(20) from the results available at the multiple defined timepoints for these variables shown in table 1, with any excess results removed.

The final extract file containing anonymised data will then be securely transferred to the University of Surrey and stored on secure research drives accessible only by the research team. Participants will not be identified or contacted during this study.

For peer review only

For peer review only - http://bmjopen.bmj.com/site/about/guidelines.xhtml

Table 1 Description of the variables to be extracted at the timepoints specified to enable calculation of ENDPAC scores

Variable name	Timepoint / time window	Details
Index glycated haemoglobin (HbA1c) result (1)	Index date	- HbA1c result in mmol/mol for those meeting the inclusion criteria
Age-group	Index date	- Participant's age-group at index date, in 5-year bands
Gender (m/f)	N/A	- Latest recorded
Height	N/A	- Latest recorded aged 18 years or older
HbA1c (2a)	Between 9 and 15 months prior to index date	- If multiple values, provide highest value
HbA1c (2b)	Between 15 and 18 months prior to index date	- If multiple values, provide highest value
HbA1c (2c)	Between 6 and 9 months prior to index date	- If multiple values, provide highest value
HbA1c (2d)	Between 3 and 6 months prior to index date	- If multiple values, provide highest value
Weight or BMI and height (1a)	Between index and 3 months after index date	- If multiple values, provide earliest value (closest to index date)
Weight or BMI and height (1b)	Between index and 3 months prior to index date	- If multiple values, provide latest value (value closest to index date)
Weight or BMI and height (2a)	Between 6 and 12 months prior to index date	- If multiple values, provide earliest value (closest to 12 months prior to index date)
Weight or BMI and height (2b)	Between 12 months and 10 years prior to index date	- If multiple values, provide latest value (closest to 12 months prior to index date)
Pancreatic cancer diagnosis	Earliest result in participant's history	- If code for 'Malignant tumour of pancreas (disorder)' or any child* codes (excluding recurrence or metastasis) is present, state whether before or after index date
Cancer antigen 19-9 (CA 19-9) result	Earliest result in participant's history	- State whether below or above threshold value and if before or after index date
Carcinoembryonic antigen (CEA) result	Earliest result in participant's history	- State whether below or above threshold value and if before or after index date

*The term 'child' refers to the relationship between the main code (the 'parent' code) and its related ('child') codes, and does not relate to the age or relationship of the participants.

For peer review only - http://bmjopen.bmj.com/site/about/guidelines.xhtml

ENPAC score calculation

For participants with the required results, ENDPAC scores will be calculated according to the process defined by ENDPAC's developers, using HbA1c mmol/mol results equivalent to the original calculator's fasting blood glucose and estimated average glucose results.(20)

Data analysis

Table 2 shows the descriptive statistics that will be used to describe the demographics of the cohort, including counts with percentages, means with standard deviations (SD) and medians with inter quartile ranges (IQR). We will assume that data are missing at random and therefore results will be calculated using the available data. The proportion of missing data will be described using counts with percentages. We will provide counts with percentages of participants for whom HbA1c, weight, BMI and height results are available and specifying for which timepoints. For the participants meeting the study's inclusion criteria, we will be able to assess the availability of data for the variables listed through the analysis summarised in Table 2.

Table 2 Details of the planned descriptive statistics for the specified variables

Variable		Descriptive statistics
Age-group	5-year bandings: 50-54, 55-59, 60-64 etc.	*n* (%)
Gender	Male	*n* (%)
	Female	
Body weight (kg), BMI and height	Result at index date	Mean (SD)
	Result ~1 year prior to index date	
	Weight change	
	Weight change	Median (IQR)
	Between index date and 3 months after index date	*n* (%)
	Between index date and 3 months prior to index date	
	Between 6 and 12 months prior to index date	
	Between 12 months and 10 years prior to index date	
HbA1c (mmol/mol)	Result at index date	Mean (SD)
	Result ~1 year prior to index date	
	HbA1c change	
	HbA1c change	Median (IQR)
	Between 15 and 18 months prior to index date	*n* (%)
	Between 15 and 18 months prior to index date	
	Between 6 and 9 months prior to index date	
	Between 3 and 6 months prior to index date	
HbA1c category* at index date	Category 5	*n* (%)
	Category 4	
HbA1c category ~1 year prior to index date	Category 3	*n* (%)
	Category 2	
	Category 1	

For peer review only

For peer review only - http://bmjopen.bmj.com/site/about/guidelines.xhtml

Change of HbA1c category		Mean (SD)
Change of HbA1c category		Median (IQR)
ENDPAC score	-6 to +11	*n* (%)
Cancer antigen 19-9 (CA 19-9) result	Available	*n* (%)
	Above threshold value	*n* (%)
Carcinoembryonic antigen (CEA) result	Available	*n* (%)
	Above threshold value	*n* (%)
Pancreatic cancer diagnosis	Available	*n* (%)
	Prior to index date	*n* (%)
	At or post index date	
*HbA1c categories are defined by the ENDPAC calculator, depending on the HbA1c levels at index date and pre-index date(30)		

We will report on the number of cases for whom ENDPAC scores can be calculated per practice, in addition to the distribution of the scores into high- (≥3), intermediate- (2–1) or low-risk (≤0) groups for pancreatic cancer at the time they first meet the glycaemic definition of new-onset diabetes,(20) and include the timepoints from which the HbA1c and weight results were taken. This will enable us to provide estimates on the number of patients who would need clinical workup for pancreatic cancer if the ENDPAC model were to be deployed across the UK, thereby assessing the potential resource burden on the NHS.

Project governance

The study will be overseen by a steering group of GPs from the Surrey and Sussex Cancer Alliance. They will meet with the study team every two months to discuss progress. The steering group and other stakeholders, including the NHS Cancer Programme strategy team, the charitable patient advocate group Pancreatic Cancer Action (PCA) and a pancreatic cancer survivor, have already been and will continue to be involved throughout the study, providing advice and guidance on study design, recruitment, and dissemination strategies.

Patient and public involvement

PCA has a well-established patient and public involvement group. Their expertise and feedback will be incorporated throughout this study and they will also support study dissemination, including publication writing and seminars.

Discussion

For ENDPAC to be of benefit to patients in the UK, the availability and quality of the required data and the means to extract it from primary care providers needs to be established, and this will be the first study the authors are aware of to do so in a UK setting. In achieving this, it will be the first to develop scalable semi-automated methods for data extraction to obtain the results required to calculate ENDPAC scores from UK primary care patient record management software platforms. In addition, the extraction methods will enable GP practice staff to validate a portion of the extracted data prior to transfer to the research team, thereby providing confidence in the findings. Through assessing the availability and quality of the data, the feasibility of rolling-out ENDPAC in UK primary care can be established, and the resource impact on the NHS estimated, based on the number of participants warranting clinical workup through sufficiently high ENDPAC scores.

Preliminary exploratory analysis undertaken by the research team of coded diabetes diagnoses in primary care records indicates that on average, approximately 30-40 patients per practice in the UK are diagnosed with new-onset diabetes annually. As the study period covers three years, and as we

For peer review only

For peer review only - http://bmjopen.bmj.com/site/about/guidelines.xhtml

are defining new-onset diabetes solely using HbA1c results rather than relying on coded diagnoses, it is estimated that each participating GP practice will provide data for at least 100 participants. Therefore, with 20 practices participating, approximately 2000 participants' records will be provided. Only anonymised data will be extracted and analysed, meaning that individual participants' ENDPAC scores will not be reported.

The quality of routine data presents a challenge in any data-driven study. For example, weight, BMI and HbA1c measurements are opportunistically collected in clinical practice, and therefore are not necessarily available at regular time intervals. Through discussion with the study's steering group, even though weight and height are needed to calculate BMI, patient record management systems do not always require the underlying values at that timepoint to be entered when recording or calculating BMI. As ENDPAC requires weight results for score calculation, we are extracting BMI and height values in addition to weight, to enable back-calculation of weight results if only BMI and height values are provided at the required timepoints. This will maximise the number of eligible participants for whom an ENDPAC score can be calculated. We will provide feedback to the practices if any particular issues are encountered with missing results in their practices, and how they might improve on this.

In this study we will use single, unpaired HbA1c results, whilst ENDPAC was originally developed for use with paired results from a combination of fasting blood glucose, random average glucose, HbA1c or oral glucose load test results.(20) This is because both external validation studies reported that participants had substantially more HbA1c results than other blood glucose measurements, and recommended that ENDPAC be applied in a real world setting to those diagnosed with diabetes through HbA1c only.(24,25) In addition, Khan *et al*.'s external validation successfully used single HbA1c results, rather than requiring paired results.(24) Furthermore, the UK stakeholder GPs involved in planning the current study have highlighted that HbA1c is their preferred means of assessing patients' blood glucose, and is the principal method for diabetes monitoring in the UK.(40) It is for these reasons that HbA1c results will be used in this study.

The three-year time window in use by this study is based on the significantly increased risk of pancreatic cancer in the three years after diagnosis of new-onset diabetes.(20,41,42) Sharma *et al*.(20) suggest that with sufficient additional case review processes for those having ENDPAC scores calculated, 50 % of false positives can be removed, increasing the 3-year risk of pancreatic cancer for patients with an ENDPAC score ≥ 3 from 3.6 % to 10 %. This process includes reviewing patients' records for other causes of weight loss, recent steroid use causing rapid blood glucose increases, and uncontrolled diabetes causing rapid weight gain pre-index and rapid weight loss post-index. For the purposes of this feasibility study, such additional case review processes are not considered necessary for inclusion within the data extraction process. This is because depending on the outcome of this feasibility study, we plan to design and deliver a clinical intervention collaborating with patients and clinicians, aiming to improve early diagnosis by using ENDPAC scores. In the future study, after clinical consultation involving manual case review by the participants' GPs to assess each participant's suitability for participation, participants with an elevated ENDPAC score will be invited for further investigations, such as blood tests and pancreatic scans, to rule out or diagnose pancreatic cancer.

Ethics and dissemination

This project has been reviewed by the University of Surrey University Ethics Committee and has received a favourable ethical opinion (FHMS 22-23 151 EGA). We will comply with the legal and policy requirements of the University of Surrey.

For peer review only - http://bmjopen.bmj.com/site/about/guidelines.xhtml

Data extracts created as part of this project will remain under the management of GP practices. Data will not be made open access or deposited in any repository, as outlined in the data sharing agreement. Subject to all necessary approvals, data may be made available for secondary use by the GP practices who remain data controllers.

Results will be presented at scientific meetings and published in international peer-reviewed journals. Summaries will be provided to the participating GP practices, clinical leads, and policy makers.

Author contributions

HC, AL, CH and JT conceived the original study idea. All authors contributed to designing and planning the study. HC and AL wrote the protocol and obtained ethics approval. HC drafted the first version of the manuscript. All authors contributed to writing and reviewing the manuscript.

Acknowledgements

The work of NPL co-authors was funded by the UK Government's Department for Science, Innovation & Technology through the UK's National Measurement System programmes.

Funding statement

This research was conducted as part of a PhD studentship funded by the University of Surrey and undertaken by HC. This study received seed funding from the Faculty of Health and Medical Sciences, University of Surrey awarded to AL.

Competing Interests statement

JT's role in Surrey and Sussex Cancer Alliance is part funded by Cancer Research UK.

References

1. Sung H, Ferlay J, Siegel RL, Laversanne M, Soerjomataram I, Jemal A, et al. Global Cancer Statistics 2020: GLOBOCAN Estimates of Incidence and Mortality Worldwide for 36 Cancers in 185 Countries. CA: A Cancer Journal for Clinicians. 2021;71(3):209–49.

2. Roberts KJ, Prasad P, Steele Y, Marcon F, Faulkner T, Cilliers H, et al. A reduced time to surgery within a 'fast track' pathway for periampullary malignancy is associated with an increased rate of pancreatoduodenectomy. HPB. 2017 Aug 1;19(8):713–20.

3. Moini J, Badolato C, Ahangari R. Chapter 15 - Pancreatic Tumors. In: Moini J, Badolato C, Ahangari R, editors. Epidemiology of Endocrine Tumors [Internet]. Elsevier; 2020 [cited 2022 Oct 16]. p. 335–58. Available from: https://www.sciencedirect.com/science/article/pii/B9780128221877000128

4. Huang L, Jansen L, Balavarca Y, Molina-Montes E, Babaei M, van der Geest L, et al. Resection of pancreatic cancer in Europe and USA: an international large-scale study highlighting large variations. Gut. 2019 Jan;68(1):130–9.

5. Afolabi PR, McDonnell D, Byrne CD, Wilding S, Goss V, Walters J, et al. DEPEND study protocol: early detection of patients with pancreatic cancer – a pilot study to evaluate the utility of faecal elastase-1 and 13C-mixed triglyceride breath test as screening tools in high-risk individuals. BMJ Open. 2022 Feb 1;12(2):e057271.

For peer review only - http://bmjopen.bmj.com/site/about/guidelines.xhtml

6. Armstrong EA, Beal EW, Chakedis J, Paredes AZ, Moris D, Pawlik TM, et al. Exosomes in Pancreatic Cancer: from Early Detection to Treatment. J Gastrointest Surg. 2018 Apr 1;22(4):737–50.

7. Debernardi S, Blyuss O, Rycyk D, Srivastava K, Jeon CY, Cai H, et al. Urine biomarkers enable pancreatic cancer detection up to 2 years before diagnosis. International Journal of Cancer. 2023;152(4):769–80.

8. Debernardi S, O'Brien H, Algahmdi AS, Malats N, Stewart GD, Plješa-Ercegovac M, et al. A combination of urinary biomarker panel and PancRISK score for earlier detection of pancreatic cancer: A case–control study. PLOS Medicine. 2020 Dec 10;17(12):e1003489.

9. Kane LE, Mellotte GS, Mylod E, O'Brien RM, O'Connell F, Buckley CE, et al. Diagnostic Accuracy of Blood-based Biomarkers for Pancreatic Cancer: A Systematic Review and Meta-analysis. Cancer Research Communications. 2022 Oct 20;2(10):1229–43.

10. Kim J, Bamlet WR, Oberg AL, Chaffee KG, Donahue G, Cao XJ, et al. Detection of early pancreatic ductal adenocarcinoma using thrombospondin-2 and CA19-9 blood markers. Sci Transl Med. 2017 Jul 12;9(398):eaah5583.

11. Schlick K, Kiem D, Greil R. Recent Advances in Pancreatic Cancer: Novel Prognostic Biomarkers and Targeted Therapy-A Review of the Literature. Biomolecules. 2021 Oct 6;11(10):1469.

12. Sollie S, Michaud DS, Sarker D, Karagiannis SN, Josephs DH, Hammar N, et al. Chronic inflammation markers are associated with risk of pancreatic cancer in the Swedish AMORIS cohort study. BMC Cancer. 2019 Aug 29;19(1):858.

13. Usher-Smith J, Emery J, Hamilton W, Griffin SJ, Walter FM. Risk prediction tools for cancer in primary care. Br J Cancer. 2015 Dec;113(12):1645–50.

14. Medina-Lara A, Grigore B, Lewis R, Peters J, Price S, Landa P, et al. Cancer diagnostic tools to aid decision-making in primary care: mixed-methods systematic reviews and cost-effectiveness analysis. Health Technology Assessment. 2020 Nov 30;24(66):1–332.

15. Santos R, Coleman HG, Cairnduff V, Kunzmann AT. Clinical Prediction Models for Pancreatic Cancer in General and At-Risk Populations: A Systematic Review. Am J Gastroenterol. 2022 Sep 21;

16. Zhao D, Weng C. Combining PubMed Knowledge and EHR Data to Develop a Weighted Bayesian Network for Pancreatic Cancer Prediction. Journal of biomedical informatics. 2011 Oct;44(5):859.

17. Muhammad W, Hart GR, Nartowt B, Farrell JJ, Johung K, Liang Y, et al. Pancreatic Cancer Prediction Through an Artificial Neural Network. Frontiers in Artificial Intelligence [Internet]. 2019 [cited 2022 Nov 6];2. Available from: https://www.frontiersin.org/articles/10.3389/frai.2019.00002

18. Lu XH, Wang L, Li H, Qian JM, Deng RX, Zhou L. Establishment of risk model for pancreatic cancer in Chinese Han population. World J Gastroenterol. 2006 Apr 14;12(14):2229–34.

19. Chen Q, Cherry DR, Nalawade V, Qiao EM, Kumar A, Lowy AM, et al. Clinical Data Prediction Model to Identify Patients With Early-Stage Pancreatic Cancer. JCO Clinical Cancer Informatics. 2021 Dec;(5):279–87.

For peer review only - http://bmjopen.bmj.com/site/about/guidelines.xhtml

For peer review only

20. Sharma A, Kandlakunta H, Nagpal SJS, Feng Z, Hoos W, Petersen GM, et al. Model to Determine Risk of Pancreatic Cancer in Patients With New-Onset Diabetes. Gastroenterology. 2018 Sep;155(3):730-739.e3.

21. Hobbs FDR, Bankhead C, Mukhtar T, Stevens S, Perera-Salazar R, Holt T, et al. Clinical workload in UK primary care: a retrospective analysis of 100 million consultations in England, 2007–14. The Lancet. 2016 Jun 4;387(10035):2323–30.

22. Elliss-Brookes L, McPhail S, Ives A, Greenslade M, Shelton J, Hiom S, et al. Routes to diagnosis for cancer – determining the patient journey using multiple routine data sets. Br J Cancer. 2012 Oct;107(8):1220–6.

23. Evans J, Chapple A, Salisbury H, Corrie P, Ziebland S. "It can't be very important because it comes and goes"—patients' accounts of intermittent symptoms preceding a pancreatic cancer diagnosis: a qualitative study. BMJ Open. 2014 Feb 18;4(2):e004215.

24. Khan S, Safarudin RF, Kupec JT. Validation of the ENDPAC model: Identifying new-onset diabetics at risk of pancreatic cancer. Pancreatology. 2021 Apr;21(3):550–5.

25. Chen W, Butler RK, Lustigova E, Chari ST, Wu BU. Validation of the Enriching New-Onset Diabetes for Pancreatic Cancer Model in a Diverse and Integrated Healthcare Setting. Dig Dis Sci. 2020 Feb;66(1):78–87.

26. Woodmansey C, McGovern AP, McCullough KA, Whyte MB, Munro NM, Correa AC, et al. Incidence, Demographics, and Clinical Characteristics of Diabetes of the Exocrine Pancreas (Type 3c): A Retrospective Cohort Study. Diabetes Care. 2017 Nov 1;40(11):1486–93.

27. Chari ST, Leibson CL, Rabe KG, Timmons LJ, Ransom J, Andrade M de, et al. Pancreatic Cancer–Associated Diabetes Mellitus: Prevalence and Temporal Association With Diagnosis of Cancer. Gastroenterology. 2008 Jan 1;134(1):95–101.

28. Sah RP, Nagpal SJS, Mukhopadhyay D, Chari ST. New insights into pancreatic cancer-induced paraneoplastic diabetes. Nature reviews Gastroenterology & hepatology. 2013 Jul;10(7):423.

29. Pannala R, Basu A, Petersen GM, Chari ST. New-onset Diabetes: A Potential Clue to the Early Diagnosis of Pancreatic Cancer. The lancet oncology. 2009 Jan;10(1):88.

30. Sharma A, Smyrk TC, Levy MJ, Topazian MA, Chari ST. Fasting Blood Glucose Levels Provide Estimate of Duration and Progression of Pancreatic Cancer Before Diagnosis. Gastroenterology. 2018 Aug 1;155(2):490-500.e2.

31. Olson SH, Xu Y, Herzog K, Saldia A, DeFilippis EM, Li P, et al. Weight Loss, Diabetes, Fatigue, and Depression Preceding Pancreatic Cancer. Pancreas. 2016 Aug;45(7):986.

32. Hue JJ, Sugumar K, Kyasaram RK, Shanahan J, Lyons J, Ocuin LM, et al. Weight Loss as an Untapped Early Detection Marker in Pancreatic and Periampullary Cancer. Ann Surg Oncol. 2021 Oct;28(11):6283–92.

33. Li D, Mao Y, Chang P, Liu C, Hassan MM, Yeung SJ, et al. Impacts of new-onset and long-term diabetes on clinical outcome of pancreatic cancer. Am J Cancer Res. 2015 Sep 15;5(10):3260–9.

For peer review only - http://bmjopen.bmj.com/site/about/guidelines.xhtml

For peer review only

34. Pannala R, Leibson CL, Rabe KG, Timmons LJ, Ransom J, de Andrade M, et al. Temporal Association of Changes in Fasting Blood Glucose and Body Mass Index with Diagnosis of Pancreatic Cancer. Am J Gastroenterol. 2009 Sep;104(9):2318–25.

35. Aggarwal G, Rabe KG, Petersen GM, Chari ST. New-onset diabetes in pancreatic cancer: A study in the primary care setting. Pancreatology. 2012;12(2):156–61.

36. Fraser LA, Twombly J, Zhu M, Long Q, Hanfelt JJ, Narayan KMV, et al. Delay in Diagnosis of Diabetes Is Not the Patient's Fault. Diabetes Care. 2010 Jan 1;33(1):e10.

37. Porta M, Curletto G, Cipullo D, Rigault de la Longrais R, Trento M, Passera P, et al. Estimating the Delay Between Onset and Diagnosis of Type 2 Diabetes From the Time Course of Retinopathy Prevalence. Diabetes Care. 2014 May 10;37(6):1668–74.

38. Bleeker SE, Moll HA, Steyerberg EW, Donders ART, Derksen-Lubsen G, Grobbee DE, et al. External validation is necessary in prediction research:: A clinical example. Journal of Clinical Epidemiology. 2003 Sep 1;56(9):826–32.

39. Price S, Spencer A, Medina-Lara A, Hamilton W. Availability and use of cancer decision-support tools: a cross-sectional survey of UK primary care. Br J Gen Pract. 2019 Jul;69(684):e437–43.

40. National Institute for Health and Care Excellence. Recommendations | Type 2 diabetes in adults: management | Guidance | NICE [Internet]. NICE; 2015 [cited 2023 Jul 31]. Available from: https://www.nice.org.uk/guidance/ng28/chapter/Recommendations#blood-glucose-management

41. Boursi B, Finkelman B, Giantonio BJ, Haynes K, Rustgi AK, Rhim AD, et al. A Clinical Prediction Model to Assess Risk for Pancreatic Cancer Among Patients With New-Onset Diabetes. Gastroenterology. 2017 Mar;152(4):840-850.e3.

42. Chari ST, Leibson CL, Rabe KG, Ransom J, de Andrade M, Petersen GM. Probability of Pancreatic Cancer Following Diabetes: A Population-Based Study. Gastroenterology. 2005 Aug 1;129(2):504–11.

For peer review only

For peer review only - http://bmjopen.bmj.com/site/about/guidelines.xhtml

BMJ Open

Determining the feasibility of calculating pancreatic cancer risk scores for people with new-onset diabetes in primary care (DEFEND PRIME): study protocol

Journal:	*BMJ Open*
Manuscript ID	bmjopen-2023-079863.R1
Article Type:	Protocol
Date Submitted by the Author:	03-Jan-2024
Complete List of Authors:	Claridge, Hugh; University of Surrey, School of Health Sciences, Faculty of Health and Medical Sciences; National Physical Laboratory<br>Price, Claire; University of Surrey, School of Health Sciences, Faculty of Health and Medical Sciences,; National Physical Laboratory<br>Ali, Rofique; Tower Hamlets Network 1 Primary Care Network<br>Cooke, Elizabeth; National Physical Laboratory<br>de Lusignan, Simon; University of Oxford, Nuffield Department of Primary Care Health Sciences<br>Harvey-Sullivan, Adam; Tower Hamlets Network 1 Primary Care Network; Queen Mary University of London, Centre for Primary Care, Wolfson Institute of Population Health<br>Hodges, Catherine; Surrey and Sussex Cancer Alliance<br>Khalaf, Natalia; Baylor College of Medicine, Center for Innovations in Quality, Effectiveness, and Safety (IQuESt), Michael E. DeBakey Veterans Affairs Medical Center, Section of Gastroenterology and Hepatology, Department of Medicine<br>O'Callaghan, Dean; Tower Hamlets Network 1 Primary Care Network<br>Stunt, Ali; Pancreatic Cancer Action<br>Thomas, Spencer; National Physical Laboratory<br>Thomson, Joanna; Surrey and Sussex Cancer Alliance<br>Lemanska, Agnieszka; University of Surrey, School of Health Sciences, Faculty of Health and Medical Sciences; National Physical Laboratory
**Primary Subject Heading**:	General practice / Family practice
Secondary Subject Heading:	Gastroenterology and hepatology, Oncology, Health services research, Diagnostics, Health informatics
Keywords:	Primary Care < Primary Health Care, Pancreatic disease < GASTROENTEROLOGY, Electronic Health Records, Feasibility Studies, Protocols & guidelines < HEALTH SERVICES ADMINISTRATION & MANAGEMENT

Determining the feasibility of calculating pancreatic cancer risk scores for people with new-onset diabetes in primary care (DEFEND PRIME): study protocol

Authors

Hugh Claridge[1,2], Claire A. Price[1,2], Rofique Ali[3], Elizabeth A. Cooke[2], Simon de Lusignan[4], Adam Harvey-Sullivan[3,5], Catherine Hodges[6], Natalia Khalaf[7], Dean O'Callaghan[3], Ali Stunt[8], Spencer A. Thomas[2], Joanna Thomson[6], Agnieszka Lemanska[1,2]

[1]School of Health Sciences, Faculty of Health and Medical Sciences, University of Surrey, Guildford, UK
[2]National Physical Laboratory, Teddington, UK
[3]Tower Hamlets Network 1 Primary Care Network, London, UK
[4]Nuffield Department of Primary Care Health Sciences, University of Oxford, Oxford, UK
[5]Centre for Primary Care, Wolfson Institute of Population Health, Queen Mary University of London
[6]Surrey and Sussex Cancer Alliance, Guildford, UK
[7]Section of Gastroenterology and Hepatology, Department of Medicine, Baylor College of Medicine, Center for Innovations in Quality, Effectiveness, and Safety (IQuESt), Michael E. DeBakey Veterans Affairs Medical Center, Houston, Texas, USA
[8]Pancreatic Cancer Action, Hampshire, UK
Correspondence to: Hugh Claridge, h.claridge@surrey.ac.uk

Abstract

Introduction

Worldwide, pancreatic cancer has a poor prognosis. Early diagnosis may improve survival by enabling curative treatment. Statistical and machine learning diagnostic prediction models using risk factors such as patient demographics and blood tests are being developed for clinical use to improve early diagnosis. One example is the Enriching New-onset Diabetes for Pancreatic Cancer (ENDPAC) model, which employs patients' age, blood glucose and weight changes to provide pancreatic cancer risk scores. These values are routinely collected in primary care in the United Kingdom (UK). Primary care's central role in cancer diagnosis makes it an ideal setting to implement ENDPAC but it has yet to be used in clinical settings. This study aims to determine the feasibility of applying ENDPAC to data held by UK primary care practices.

Methods and analysis

This will be a multi-centre observational study with a cohort design, determining the feasibility of applying ENDPAC in UK primary care. We will develop software to search, extract and process anonymised data from 20 primary care providers' electronic patient record management systems on participants aged 50+ years, with a glycated haemoglobin (HbA1c) test result of ≥ 48 mmol/mol (6.5 %) and no previous abnormal HbA1c results. Software to calculate ENDPAC scores will be developed, and descriptive statistics used to summarise the cohort's demographics and assess data quality. Findings will inform the development of a future UK clinical trial to test ENDPAC's effectiveness for the early detection of pancreatic cancer.

Ethics and dissemination

This project has been reviewed by the University of Surrey University Ethics Committee and received a favourable ethical opinion (FHMS 22-23 151 EGA). Study findings will be presented at scientific

For peer review only - http://bmjopen.bmj.com/site/about/guidelines.xhtml

meetings and published in international peer-reviewed journals. Participating primary care practices, clinical leads and policy makers will be provided with summaries of the findings.

Strengths and limitations of this study's methods

- Early computerisation of UK primary care, incorporating linkage to pathology systems combined with pay-for-performance for chronic disease management including diabetes, helps to ensure population-wide data.
- The extraction software will permit validation of the extracted data by primary care staff prior to transfer to the research team.
- Using HbA1c results only to define new-onset diabetes means this study is not impacted by the quality of diabetes diagnosis coding in primary care.
- This study will raise awareness of new-onset diabetes' association with pancreatic cancer within the primary care community.
- The study period includes the COVID-19 pandemic, thus the data within this period may not reflect data obtained before or after the pandemic.

Introduction

Pancreatic cancer and early diagnosis

Pancreatic cancer is the seventh leading cause of global cancer deaths, with only 10–20 % of patients diagnosed at a sufficiently early stage for curative intervention.(1,2) Survival can be dramatically improved if diagnosed earlier, at a local rather than distant stage – 37 % versus 3 % 5-year survival rate respectively.(3,4) However, there are multiple barriers to early diagnosis including the non-specific nature of the early symptoms,(5) and lack of suitable diagnostic biomarkers, although advances are being made in this area.(6–12)

As with other health conditions including cancers of other sites,(13) statistical and machine learning clinical prediction models are being developed for clinical use, to facilitate earlier diagnosis of pancreatic cancer, in particular its most common subtype, pancreatic ductal adenocarcinoma.(3,14,15) These models range widely in complexity,(16–20) with the simplest models including only a few variables that can also be routinely collected in primary care, making them potentially feasible for use in this setting.

The role of primary care in the UK

In most developed countries, primary care is central to healthcare provision; in the UK, 90 % of contacts with the National Health Service (NHS) are through primary care.(21) Primary care providers, including general practitioners (GPs), play a central role in assessing and addressing patients' cancer risk.(22) However, it is estimated that GPs see only one new case of pancreatic cancer every five years(23) and, when combined with the non-specific nature of its early symptoms, detection can be very difficult. Clinical diagnostic prediction models are therefore of real potential value for these clinicians, especially given these challenges of diagnosing pancreatic cancer in the context of their busy work schedules.

Enriching New-Onset Diabetes for Pancreatic Cancer (ENDPAC) model

The simplicity of the ENDPAC model makes it ideally suited for use in primary care as it only uses patient age, weight change and blood glucose measurements, which are routinely collected.(20,24,25) The model is based on the well-documented association of pancreatic cancer with older age and the paradoxical development of diabetes with weight loss.(26–34) It also captures the more rapid onset of glycaemic dysregulation found in pancreatic cancer-related diabetes than found in type 2 diabetes. As

For peer review only - http://bmjopen.bmj.com/site/about/guidelines.xhtml

the clinical diagnosis of diabetes sometimes occurs months or even years after diabetes onset,(35–37) ENDPAC instead uses the biochemically-detected glycaemic onset. This avoids these potential delays thereby maximising new-onset diabetes' potential for the early diagnosis of pancreatic cancer.(20) It therefore means that those deemed by the model as having new-onset diabetes, may not otherwise be medically diagnosed as having diabetes, but rather hyperglycaemia. The model has undergone external validation in three separate studies, two using data from the United States (US) and one using data from Israel, establishing that ENDPAC demonstrates a reasonable ability to differentiate patients with type 2 diabetes from those with glycaemia-defined diabetes who later develop pancreatic cancer.(24,25,38) It is for these reasons that this study will investigate ENDPAC's feasibility for use in UK primary care settings.

ENDPAC scores

ENDPAC calculates risk scores that patients have pancreatic cancer, by using their age and changes over time to their weight and blood glucose results. According to the model's developers, a score ≤ 0 has a sufficiently high negative predictive value for pancreatic cancer that those with this score can be deemed as only needing management for type 2 diabetes, given their very low risk of pancreatic cancer. A score ≥ 3 is considered to warrant clinical workup for pancreatic cancer.(20) This is because in the original development study and three subsequent external validation studies, patients with a score ≥ 3 had, respectively, a 3.6 %, 2.0 %, 2.6 % and 2.6 % 3-year risk of pancreatic cancer, with sensitivities of 78 %, 63 %, 42 % and 54 %.(20,25,24,38). The reduced performance in the external validation studies is unsurprising, as performance is often lower when models are applied to different populations than those used to build the model.(39) Furthermore, Sharma *et al*.(20) suggest that with sufficient additional case review processes, 50 % of false positives can be removed, increasing the 3-year risk of pancreatic cancer for patients with a score ≥ 3 from 3.6 % to 10 %.

Data extraction and value selection software

In order to calculate ENDPAC scores, specific results for HbA1c, weight or BMI need to be obtained within defined time periods to quantify the changes in these results over time.(20) The process of manually searching patient record management systems for participants meeting the inclusion criteria and extracting the correct results for every individual would be extremely time-consuming. In addition to this, manually performing this process, or permitting the participating primary care practices to develop their own mechanisms for doing so, would bring into question the reliability and accuracy of the results extracted for each individual as the value selection criteria are extremely complex to apply. This could potentially undermine the validity of the findings of this feasibility study.

To address these issues, we will develop and provide software to primary care practices for the UK's two main primary care patient record management systems which are used by over 85 % of primary care providers: EMIS Web by EMIS Health and SystmOne by TPP.(40) The software will search the electronic healthcare records within these patient record management systems, extract the data for those meeting the inclusion criteria, and apply complex value selection procedures to obtain the data needed to calculate ENDPAC scores. This will ensure the consistency, reproducibility and reliability of the data extracts as well as use as little of the practice staff's time as possible, thereby increasing the potential usability of ENDPAC in clinical settings.

Rationale for this feasibility study

The ENDPAC model was developed using data from the US.(20) However, to date, ENDPAC has not been reported as tested in the UK. Whilst patient weight and blood glucose measures are routinely collected in many developed nations, there may be different approaches to gathering these data and different units of measurement used both between and within countries. Therefore, through the

For peer review only - http://bmjopen.bmj.com/site/about/guidelines.xhtml

development of data extraction, value selection and ENDPAC score calculation software, we will be investigating the availability and quality of these data. This is a critically important step to undertake before considering using ENDPAC in UK clinical practice, as these aspects will directly impact whether ENDPAC scores can be calculated using UK primary care data.

Study aim and objectives

The aim is to determine the feasibility of calculating ENDPAC scores for people with new-onset diabetes in UK primary care practices.

The objectives are to:

1. Develop data extraction and value selection software for primary care. We will work with software developers for primary care patient record management systems to develop and test the software for data extraction and value selection.
2. Extract data from 20 primary care practices and evaluate the availability and quality of data.
3. Develop ENDPAC score calculation software and undertake descriptive data analysis. We will report the number of people with ENDPAC scores warranting referral for pancreatic cancer investigations and their clinical and demographic characteristics.

Methods and analysis

Design and setting

Determining the feasibility of calculating pancreatic cancer risk scores for people with new-onset diabetes in primary care (DEFEND PRIME) will be a multi-centre observational study with a cohort design in the UK. We will extract anonymised data from 20 primary care practices for people with new-onset diabetes identified within the most recent three-year period.

Primary care practice recruitment

We will use several recruitment strategies:

- Presentations and networking at conferences and meetings attended by clinicians and academics working in the early detection field.
- Newsletters from relevant charities and clinical governing bodies.
- Advertising on social media channels.
- Dissemination of study information by stakeholders and colleagues through professional networks.

Practices will enrol by completing a data sharing agreement. Based on an hourly rate of £50 for an estimated seven hours' work to extract the data, each practice will be reimbursed £350.

Participant eligibility

Participants must be at least 50 years old and with new-onset diabetes identified in the last three years. To be in accordance with ENDPAC, for the purposes of this study new-onset diabetes will be defined by an abnormal glycaemic test result of HbA1c ≥48 mmol/mol (6.5 %). All prior HbA1c test results for the participants must be below this level.

Data extraction and value selection software

We will develop the data extraction software with software developers who specialise in creating searches in patient record management systems. The software developed and provided to primary care practice staff will include detailed instructions to enable staff to run the data extraction and value selection software.

For peer review only - http://bmjopen.bmj.com/site/about/guidelines.xhtml

Table 1 details the data that will be extracted, which is modified from Sharma *et al*.(20) Prior to transfer to the research team, the value selection software will select the preferred HbA1c, weight and BMI results according to the priorities defined by Sharma *et al*.(20) from the results available at the multiple defined timepoints for these variables shown in table 1, with any excess results removed. The software will be piloted prior to use to ensure accuracy, with spot checks undertaken by selected primary care staff on extracted data.

The final extract file containing anonymised data will then be securely transferred to the University of Surrey and stored on secure research drives accessible only by the research team. Participants will not be identified or contacted during this study. Figure 1 shows the various steps will be undertaken following the enrolment of a primary care practice in the study, including the running of the data extraction software, value selection software and transfer of the extracted data to the study team.

[Insert Figure 1 Flow chart providing an overview of the main steps for the participating primary care practices in the DEFEND PRIME study]

For peer review only

Table 1 Description of the variables to be extracted at the timepoints specified to enable calculation of ENDPAC scores

Variable name	Timepoint / time window	Details
Index glycated haemoglobin (HbA1c) result (1)	Index date	- HbA1c result in mmol/mol for those meeting the inclusion criteria
Age-group	Index date	- Participant's age-group at index date, in 5-year bands
Gender (m/f)	N/A	- Latest recorded
Height	N/A	- Latest recorded aged 18 years or older
HbA1c (2a)	Between 9 and 15 months prior to index date	- If multiple values, provide highest value
HbA1c (2b)	Between 15 and 18 months prior to index date	- If multiple values, provide highest value
HbA1c (2c)	Between 6 and 9 months prior to index date	- If multiple values, provide highest value
HbA1c (2d)	Between 3 and 6 months prior to index date	- If multiple values, provide highest value
HbA1c (2e)	Between 18 and 24 months prior to index date	- If multiple values, provide highest value
Weight or BMI and height (1a)	Between index and 3 months after index date	- If multiple values, provide earliest value (closest to index date)
Weight or BMI and height (1b)	Between index and 3 months prior to index date	- If multiple values, provide latest value (closest to index date)
Weight or BMI and height (2a)	Between 9 and 12 months prior to index date	- If multiple values, provide earliest value (closest to 12 months prior to index date)
Weight or BMI and height (2b)	Between 12 and 15 months prior to index date	- If multiple values, provide latest value (closest to 12 months prior to index date)
Weight or BMI and height (2c)	Between 15 and 18 months prior to index date	- If multiple values, provide latest value (closest to 15 months prior to index date)
Weight or BMI and height (2d)	Between 3 and 9 months prior to index date	- If multiple values, provide earliest value (closest to 9 months prior to index date)
Weight or BMI and height (2e)	Between 18 months and 10 years prior to index date	- If multiple values, provide latest value (closest to 18 months prior to index date)

For peer review only - http://bmjopen.bmj.com/site/about/guidelines.xhtml

Pancreatic cancer diagnosis	Earliest result in participant's history	- If code for 'Malignant tumour of pancreas (disorder)' or any child* codes (excluding recurrence or metastasis) is present, state whether before or after index date
Cancer antigen 19-9 (CA 19-9) result	Earliest result in participant's history	- State whether below or above threshold value and if before or after index date
Carcinoembryonic antigen (CEA) result	Earliest result in participant's history	- State whether below or above threshold value and if before or after index date
*The term 'child' refers to the relationship between the main code (the 'parent' code) and its related ('child') codes, and does not relate to the age or relationship of the participants.		

For peer review only

For peer review only - http://bmjopen.bmj.com/site/about/guidelines.xhtml

ENPAC score calculation

For participants with the required results, ENDPAC scores will be calculated by the research team using the score calculation software being developed, according to the process defined by ENDPAC's developers. This software will use HbA1c mmol/mol results equivalent to the original calculator's fasting blood glucose and estimated average glucose results.(20)

Data analysis

Table 2 shows the descriptive statistics that will be used to describe the demographics of the cohort, including counts with percentages, means with standard deviations (SD) and medians with inter quartile ranges (IQR). We will assume that data are missing at random and therefore results will be calculated using the available data. The proportion of missing data will be described using counts with percentages. We will provide counts with percentages of participants for whom HbA1c, weight, BMI and height results are available and specifying for which timepoints. For the participants meeting the study's inclusion criteria, we will be able to assess the availability of data for the variables listed through the analysis summarised in Table 2.

Table 2 Details of the planned descriptive statistics for the specified variables

Variable		Descriptive statistics
Age-group	5-year bandings: 50-54, 55-59, 60-64 etc.	*n* (%)
Gender	Male	*n* (%)
	Female	
Body weight (kg), BMI and height	Result at index date	Mean (SD)
	Result ~1 year prior to index date	
	Weight change	
	Weight change	Median (IQR)
	Between index date and 3 months after index date	*n* (%)
	Between index date and 3 months prior to index date	
	Between 9 and 12 months prior to index date	
	Between 12 and 15 months prior to index date	
	Between 15 and 18 months prior to index date	
	Between 3 and 9 months prior to index date	
	Between 18 months and 10 years prior to index date	
HbA1c (mmol/mol)	Result at index date	Mean (SD)
	Result ~1 year prior to index date	
	HbA1c change	
	HbA1c change	Median (IQR)
	Between 9 and 15 months prior to index date	*n* (%)
	Between 15 and 18 months prior to index date	
	Between 6 and 9 months prior to index date	

For peer review only - http://bmjopen.bmj.com/site/about/guidelines.xhtml

	Between 3 and 6 months prior to index date	
	Between 18 and 24 months prior to index date	
HbA1c category* at index date	Category 5	*n* (%)
	Category 4	
HbA1c category ~1 year prior to index date	Category 3	*n* (%)
	Category 2	
	Category 1	
Change of HbA1c category		Mean (SD)
Change of HbA1c category		Median (IQR)
ENDPAC score	-6 to +11	*n* (%)
Cancer antigen 19-9 (CA 19-9) result	Available	*n* (%)
	Above threshold value	*n* (%)
Carcinoembryonic antigen (CEA) result	Available	*n* (%)
	Above threshold value	*n* (%)
Pancreatic cancer diagnosis	Available	*n* (%)
	Prior to index date	*n* (%)
	At or post index date	

*HbA1c categories are defined by the ENDPAC calculator, depending on the HbA1c levels at index date and pre-index date(30)

We will report on the number of cases for whom ENDPAC scores can be calculated per practice, in addition to the distribution of the scores into high- (≥3), intermediate- (2–1) or low-risk (≤0) groups for pancreatic cancer at the time they first meet the glycaemic definition of new-onset diabetes,(20) and include the timepoints from which the HbA1c and weight results were taken. This will enable us to provide estimates on the number of patients who would need clinical workup for pancreatic cancer if the ENDPAC model were to be deployed across the UK, thereby assessing the potential resource burden on the NHS.

Project governance

The study will be overseen by a steering group of GPs from the Surrey and Sussex Cancer Alliance. They will meet with the study team every two months to discuss progress. The steering group and other stakeholders, including the NHS Cancer Programme strategy team and the charitable patient advocate group Pancreatic Cancer Action have already been and will continue to be involved throughout the study, providing advice and guidance on study design, recruitment, and dissemination strategies.

Patient and public involvement and engagement

We have been working closely with a pancreatic cancer survivor, who was involved in the conception of the study and this protocol's development. They contribute to the project through the regular two-monthly project meetings. They are updated on progress and have input on the study's design, conduct and recruitment processes. In the future, they will be involved in dissemination. We are also working with two charities, Pancreatic Cancer Action and Pancreatic Cancer UK, who have well-established patient and public involvement groups, including pancreatic cancer survivors and family members of those with the disease. We will consult these groups during study delivery and dissemination. Their expertise and feedback will be incorporated throughout this study and they will also support study dissemination, including involvement in conference presentations, webinars and publications as well as in developing plain English summaries.

For peer review only

For peer review only - http://bmjopen.bmj.com/site/about/guidelines.xhtml

Discussion

This will be the first study the authors are aware of to determine whether ENDPAC scores can be calculated for patients in UK primary care. We will achieve this by developing scalable software that will extract the required data, conduct the complex value selection process and calculate ENDPAC scores. This will enable the assessment of the availability and quality of the data required for score calculation, in addition to enabling primary care practice staff to validate a portion of the extracted data prior to transfer to the research team thus providing confidence in the findings. Through assessing the availability and quality of the data, the feasibility of rolling-out ENDPAC in UK primary care can be established, and the resource impact on the NHS estimated, based on the number of participants warranting clinical workup through sufficiently high ENDPAC scores.

The quality of routine data presents a challenge in any data-driven study. For example, weight, BMI and HbA1c measurements are opportunistically collected in clinical practice, and therefore are not necessarily available at regular time intervals. Through discussion with the study's steering group, even though weight and height are needed to calculate BMI, patient record management systems do not always require the underlying values at that timepoint to be entered when recording or calculating BMI. As ENDPAC requires weight results for score calculation, we are extracting BMI and height values in addition to weight, to enable back-calculation of weight results if only BMI and height values are provided at the required timepoints. This will maximise the number of eligible participants for whom an ENDPAC score can be calculated. We will provide feedback to the practices if any particular issues are encountered with missing results in their practices, and how they might improve on this.

Given the aims of this is feasibility study, no sample size calculation was performed.(41) The chosen sample size is pragmatic and based on the findings of preliminary exploratory analysis undertaken by the research team of coded diabetes diagnoses in primary care records. This indicated that on average, approximately 30-40 patients per practice in the UK are diagnosed with new-onset diabetes annually. As the study period covers three years, and as we are defining new-onset diabetes solely using HbA1c results rather than relying on coded diagnoses, it is estimated that each participating primary care practice will provide data for at least 100 participants. Therefore, with 20 practices participating, approximately 2000 participants' records will be provided and we regard this to be suitable to achieve this feasibility study's aims. Only anonymised data will be extracted and analysed, meaning that individual participants' ENDPAC scores will not be reported.

The study period includes the COVID-19 pandemic, thus the data extracted may not reflect data obtained before or after the pandemic. This is because patients' healthcare-seeking behaviour changed during the pandemic, resulting in atypical fluctuations in attendance to UK healthcare settings, including primary care.(42) Therefore, it may be that fewer HbA1c and weight results will be present in the participants' records in the pandemic period, and this will be taken into consideration.

In this study we will use single, unpaired HbA1c results, whilst ENDPAC was originally developed for use with paired results from a combination of fasting blood glucose, random blood glucose, HbA1c or oral glucose load test results.(20) This is because the external validation studies reported that participants had substantially more HbA1c results than other blood glucose measurements, and recommended that ENDPAC be applied in a real world setting to those diagnosed with diabetes through HbA1c only.(24,25) In addition, Khan *et al.*'s external validation successfully used single HbA1c results, rather than requiring paired results.(24) Furthermore, the UK stakeholder GPs involved in planning the current study have highlighted that HbA1c is their preferred means of

For peer review only - http://bmjopen.bmj.com/site/about/guidelines.xhtml

assessing patients' blood glucose, and is the principal method for diabetes monitoring in the UK.(43) It is for these reasons that HbA1c results will be used in this study.

The three-year time window we are using in this study is based on the significantly increased risk of pancreatic cancer in the three years after diagnosis of new-onset diabetes.(20,44,45) Sharma *et al*.(20) suggest that with sufficient additional case review processes for those having ENDPAC scores calculated, 50 % of false positives can be removed, increasing the 3-year risk of pancreatic cancer for patients with an ENDPAC score ≥ 3 from 3.6 % to 10 %. This process includes reviewing patients' records for other causes of weight loss, recent steroid use causing rapid blood glucose increases, and uncontrolled diabetes causing rapid weight gain pre-index and rapid weight loss post-index. For the purposes of this feasibility study, such additional case review processes are not considered necessary for inclusion within the data extraction process. This is because depending on the outcome of this feasibility study, we plan to design and deliver a clinical intervention collaborating with patients and clinicians, aiming to improve early diagnosis by using ENDPAC scores. In the future study, after clinical consultation involving manual case review by clinicians to assess each participant's suitability for participation, participants with an elevated ENDPAC score will be invited for further investigations, such as blood tests and pancreatic scans, to rule out or diagnose pancreatic cancer.

Ethics and dissemination

This project has been reviewed by the University of Surrey University Ethics Committee and has received a favourable ethical opinion (FHMS 22-23 151 EGA). We will comply with the legal and policy requirements of the University of Surrey.

Data extracts created as part of this project will remain under the management of primary care practices. Data will not be made open access or deposited in any repository, as outlined in the data sharing agreement. Subject to all necessary approvals, data may be made available for secondary use by the primary care practices who remain data controllers.

Results will be presented at scientific meetings and published in international peer-reviewed journals. Summaries will be provided to the participating primary care practices, clinical leads, and policy makers.

Author contributions

HC, AL, CH and JT conceived the original study idea. HC, CAP, RA, EAC, SdL, AH-S, CH, NK, DO'C, AS, SAT, JT and AL contributed to designing and planning the study. HC and AL wrote the protocol and obtained ethics approval. HC drafted the first version of the manuscript. HC, CAP, RA, EAC, SdL, AH-S, CH, NK, DO'C, AS, SAT, JT and AL contributed to writing and reviewing the manuscript.

Acknowledgements

The work of NPL co-authors was funded by the UK Government's Department for Science, Innovation & Technology through the UK's National Measurement System programmes.

Funding statement

This research was conducted as part of a PhD studentship funded by the University of Surrey and undertaken by HC (Grant no.: NA). This study received seed funding from the Faculty of Health and Medical Sciences, University of Surrey awarded to AL (Grant no.: NA).

For peer review only

Competing Interests statement

JT's role in Surrey and Sussex Cancer Alliance is part funded by Cancer Research UK.

References

1. Sung H, Ferlay J, Siegel RL, Laversanne M, Soerjomataram I, Jemal A, et al. Global Cancer Statistics 2020: GLOBOCAN Estimates of Incidence and Mortality Worldwide for 36 Cancers in 185 Countries. CA: A Cancer Journal for Clinicians. 2021;71(3):209–49.

2. Roberts KJ, Prasad P, Steele Y, Marcon F, Faulkner T, Cilliers H, et al. A reduced time to surgery within a 'fast track' pathway for periampullary malignancy is associated with an increased rate of pancreatoduodenectomy. HPB. 2017 Aug 1;19(8):713–20.

3. Moini J, Badolato C, Ahangari R. Chapter 15 - Pancreatic Tumors. In: Moini J, Badolato C, Ahangari R, editors. Epidemiology of Endocrine Tumors [Internet]. Elsevier; 2020 [cited 2022 Oct 16]. p. 335–58. Available from: https://www.sciencedirect.com/science/article/pii/B9780128221877000128

4. Huang L, Jansen L, Balavarca Y, Molina-Montes E, Babaei M, van der Geest L, et al. Resection of pancreatic cancer in Europe and USA: an international large-scale study highlighting large variations. Gut. 2019 Jan;68(1):130–9.

5. Afolabi PR, McDonnell D, Byrne CD, Wilding S, Goss V, Walters J, et al. DEPEND study protocol: early detection of patients with pancreatic cancer – a pilot study to evaluate the utility of faecal elastase-1 and 13C-mixed triglyceride breath test as screening tools in high-risk individuals. BMJ Open. 2022 Feb 1;12(2):e057271.

6. Armstrong EA, Beal EW, Chakedis J, Paredes AZ, Moris D, Pawlik TM, et al. Exosomes in Pancreatic Cancer: from Early Detection to Treatment. J Gastrointest Surg. 2018 Apr 1;22(4):737–50.

7. Debernardi S, Blyuss O, Rycyk D, Srivastava K, Jeon CY, Cai H, et al. Urine biomarkers enable pancreatic cancer detection up to 2 years before diagnosis. International Journal of Cancer. 2023;152(4):769–80.

8. Debernardi S, O'Brien H, Algahmdi AS, Malats N, Stewart GD, Plješa-Ercegovac M, et al. A combination of urinary biomarker panel and PancRISK score for earlier detection of pancreatic cancer: A case–control study. PLOS Medicine. 2020 Dec 10;17(12):e1003489.

9. Kane LE, Mellotte GS, Mylod E, O'Brien RM, O'Connell F, Buckley CE, et al. Diagnostic Accuracy of Blood-based Biomarkers for Pancreatic Cancer: A Systematic Review and Meta-analysis. Cancer Research Communications. 2022 Oct 20;2(10):1229–43.

10. Kim J, Bamlet WR, Oberg AL, Chaffee KG, Donahue G, Cao XJ, et al. Detection of early pancreatic ductal adenocarcinoma using thrombospondin-2 and CA19-9 blood markers. Sci Transl Med. 2017 Jul 12;9(398):eaah5583.

11. Schlick K, Kiem D, Greil R. Recent Advances in Pancreatic Cancer: Novel Prognostic Biomarkers and Targeted Therapy-A Review of the Literature. Biomolecules. 2021 Oct 6;11(10):1469.

12. Sollie S, Michaud DS, Sarker D, Karagiannis SN, Josephs DH, Hammar N, et al. Chronic inflammation markers are associated with risk of pancreatic cancer in the Swedish AMORIS cohort study. BMC Cancer. 2019 Aug 29;19(1):858.

For peer review only - http://bmjopen.bmj.com/site/about/guidelines.xhtml

13. Usher-Smith J, Emery J, Hamilton W, Griffin SJ, Walter FM. Risk prediction tools for cancer in primary care. Br J Cancer. 2015 Dec;113(12):1645–50.

14. Medina-Lara A, Grigore B, Lewis R, Peters J, Price S, Landa P, et al. Cancer diagnostic tools to aid decision-making in primary care: mixed-methods systematic reviews and cost-effectiveness analysis. Health Technology Assessment. 2020 Nov 30;24(66):1–332.

15. Santos R, Coleman HG, Cairnduff V, Kunzmann AT. Clinical Prediction Models for Pancreatic Cancer in General and At-Risk Populations: A Systematic Review. Am J Gastroenterol. 2022 Sep 21;

16. Zhao D, Weng C. Combining PubMed Knowledge and EHR Data to Develop a Weighted Bayesian Network for Pancreatic Cancer Prediction. Journal of biomedical informatics. 2011 Oct;44(5):859.

17. Muhammad W, Hart GR, Nartowt B, Farrell JJ, Johung K, Liang Y, et al. Pancreatic Cancer Prediction Through an Artificial Neural Network. Frontiers in Artificial Intelligence [Internet]. 2019 [cited 2022 Nov 6];2. Available from: https://www.frontiersin.org/articles/10.3389/frai.2019.00002

18. Lu XH, Wang L, Li H, Qian JM, Deng RX, Zhou L. Establishment of risk model for pancreatic cancer in Chinese Han population. World J Gastroenterol. 2006 Apr 14;12(14):2229–34.

19. Chen Q, Cherry DR, Nalawade V, Qiao EM, Kumar A, Lowy AM, et al. Clinical Data Prediction Model to Identify Patients With Early-Stage Pancreatic Cancer. JCO Clinical Cancer Informatics. 2021 Dec;(5):279–87.

20. Sharma A, Kandlakunta H, Nagpal SJS, Feng Z, Hoos W, Petersen GM, et al. Model to Determine Risk of Pancreatic Cancer in Patients With New-Onset Diabetes. Gastroenterology. 2018 Sep;155(3):730-739.e3.

21. Hobbs FDR, Bankhead C, Mukhtar T, Stevens S, Perera-Salazar R, Holt T, et al. Clinical workload in UK primary care: a retrospective analysis of 100 million consultations in England, 2007–14. The Lancet. 2016 Jun 4;387(10035):2323–30.

22. Elliss-Brookes L, McPhail S, Ives A, Greenslade M, Shelton J, Hiom S, et al. Routes to diagnosis for cancer – determining the patient journey using multiple routine data sets. Br J Cancer. 2012 Oct;107(8):1220–6.

23. Evans J, Chapple A, Salisbury H, Corrie P, Ziebland S. "It can't be very important because it comes and goes"—patients' accounts of intermittent symptoms preceding a pancreatic cancer diagnosis: a qualitative study. BMJ Open. 2014 Feb 18;4(2):e004215.

24. Khan S, Safarudin RF, Kupec JT. Validation of the ENDPAC model: Identifying new-onset diabetics at risk of pancreatic cancer. Pancreatology. 2021 Apr;21(3):550–5.

25. Chen W, Butler RK, Lustigova E, Chari ST, Wu BU. Validation of the Enriching New-Onset Diabetes for Pancreatic Cancer Model in a Diverse and Integrated Healthcare Setting. Dig Dis Sci. 2020 Feb;66(1):78–87.

26. Woodmansey C, McGovern AP, McCullough KA, Whyte MB, Munro NM, Correa AC, et al. Incidence, Demographics, and Clinical Characteristics of Diabetes of the Exocrine Pancreas (Type 3c): A Retrospective Cohort Study. Diabetes Care. 2017 Nov 1;40(11):1486–93.

For peer review only - http://bmjopen.bmj.com/site/about/guidelines.xhtml

27. Chari ST, Leibson CL, Rabe KG, Timmons LJ, Ransom J, Andrade M de, et al. Pancreatic Cancer–Associated Diabetes Mellitus: Prevalence and Temporal Association With Diagnosis of Cancer. Gastroenterology. 2008 Jan 1;134(1):95–101.

28. Sah RP, Nagpal SJS, Mukhopadhyay D, Chari ST. New insights into pancreatic cancer-induced paraneoplastic diabetes. Nature reviews Gastroenterology & hepatology. 2013 Jul;10(7):423.

29. Pannala R, Basu A, Petersen GM, Chari ST. New-onset Diabetes: A Potential Clue to the Early Diagnosis of Pancreatic Cancer. The lancet oncology. 2009 Jan;10(1):88.

30. Sharma A, Smyrk TC, Levy MJ, Topazian MA, Chari ST. Fasting Blood Glucose Levels Provide Estimate of Duration and Progression of Pancreatic Cancer Before Diagnosis. Gastroenterology. 2018 Aug 1;155(2):490-500.e2.

31. Olson SH, Xu Y, Herzog K, Saldia A, DeFilippis EM, Li P, et al. Weight Loss, Diabetes, Fatigue, and Depression Preceding Pancreatic Cancer. Pancreas. 2016 Aug;45(7):986.

32. Hue JJ, Sugumar K, Kyasaram RK, Shanahan J, Lyons J, Ocuin LM, et al. Weight Loss as an Untapped Early Detection Marker in Pancreatic and Periampullary Cancer. Ann Surg Oncol. 2021 Oct;28(11):6283–92.

33. Li D, Mao Y, Chang P, Liu C, Hassan MM, Yeung SJ, et al. Impacts of new-onset and long-term diabetes on clinical outcome of pancreatic cancer. Am J Cancer Res. 2015 Sep 15;5(10):3260–9.

34. Pannala R, Leibson CL, Rabe KG, Timmons LJ, Ransom J, de Andrade M, et al. Temporal Association of Changes in Fasting Blood Glucose and Body Mass Index with Diagnosis of Pancreatic Cancer. Am J Gastroenterol. 2009 Sep;104(9):2318–25.

35. Aggarwal G, Rabe KG, Petersen GM, Chari ST. New-onset diabetes in pancreatic cancer: A study in the primary care setting. Pancreatology. 2012;12(2):156–61.

36. Fraser LA, Twombly J, Zhu M, Long Q, Hanfelt JJ, Narayan KMV, et al. Delay in Diagnosis of Diabetes Is Not the Patient's Fault. Diabetes Care. 2010 Jan 1;33(1):e10.

37. Porta M, Curletto G, Cipullo D, Rigault de la Longrais R, Trento M, Passera P, et al. Estimating the Delay Between Onset and Diagnosis of Type 2 Diabetes From the Time Course of Retinopathy Prevalence. Diabetes Care. 2014 May 10;37(6):1668–74.

38. Boursi B, Patalon T, Webb M, Margalit O, Beller T, Yang YX, et al. Validation of the Enriching New-Onset Diabetes for Pancreatic Cancer Model: A Retrospective Cohort Study Using Real-World Data. Pancreas. 2022 Feb;51(2):196.

39. Bleeker SE, Moll HA, Steyerberg EW, Donders ART, Derksen-Lubsen G, Grobbee DE, et al. External validation is necessary in prediction research:: A clinical example. Journal of Clinical Epidemiology. 2003 Sep 1;56(9):826–32.

40. Price S, Spencer A, Medina-Lara A, Hamilton W. Availability and use of cancer decision-support tools: a cross-sectional survey of UK primary care. Br J Gen Pract. 2019 Jul;69(684):e437–43.

41. Billingham SA, Whitehead AL, Julious SA. An audit of sample sizes for pilot and feasibility trials being undertaken in the United Kingdom registered in the United Kingdom Clinical Research Network database. BMC Med Res Methodol. 2013 Aug 20;13:104.

For peer review only - http://bmjopen.bmj.com/site/about/guidelines.xhtml

42. Ferraro CF, Findlater L, Morbey R, Hughes HE, Harcourt S, Hughes TC, et al. Describing the indirect impact of COVID-19 on healthcare utilisation using syndromic surveillance systems. BMC Public Health. 2021 Nov 5;21(1):2019.

43. National Institute for Health and Care Excellence. Recommendations | Type 2 diabetes in adults: management | Guidance | NICE [Internet]. NICE; 2015 [cited 2023 Jul 31]. Available from: https://www.nice.org.uk/guidance/ng28/chapter/Recommendations#blood-glucose-management

44. Boursi B, Finkelman B, Giantonio BJ, Haynes K, Rustgi AK, Rhim AD, et al. A Clinical Prediction Model to Assess Risk for Pancreatic Cancer Among Patients With New-Onset Diabetes. Gastroenterology. 2017 Mar;152(4):840-850.e3.

45. Chari ST, Leibson CL, Rabe KG, Ransom J, de Andrade M, Petersen GM. Probability of Pancreatic Cancer Following Diabetes: A Population-Based Study. Gastroenterology. 2005 Aug 1;129(2):504–11.

For peer review only

For peer review only - http://bmjopen.bmj.com/site/about/guidelines.xhtml

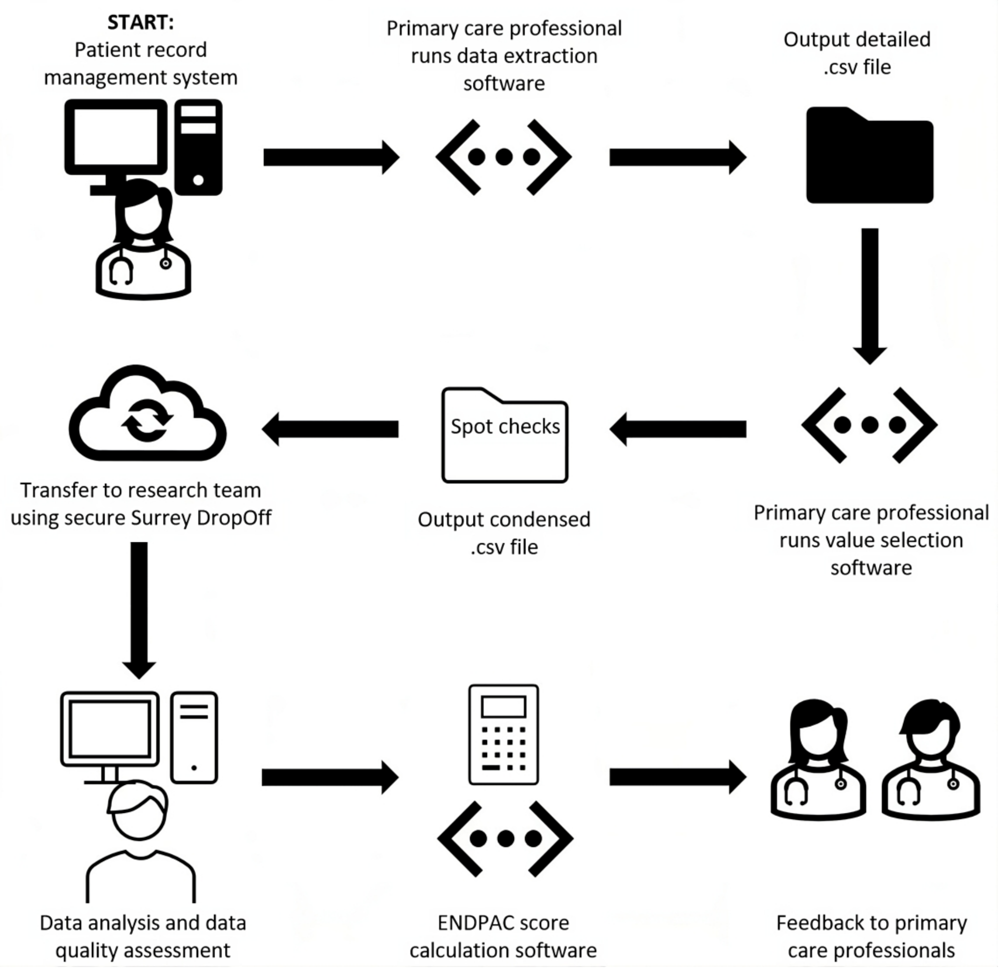

Flow chart providing an overview of the main steps for the participating primary care practices in the DEFEND PRIME study

393x379mm (300 x 300 DPI)
